# On the role of deep learning model complexity in adversarial robustness for medical images

**DOI:** 10.1186/s12911-022-01891-w

**Published:** 2022-06-20

**Authors:** David Rodriguez, Tapsya Nayak, Yidong Chen, Ram Krishnan, Yufei Huang

**Affiliations:** 1 Department of Electrical and Computer Engineering, University of Texas at San Antonio, San Antonio, TX USA; 2 Greehey Children’s Cancer Research Institute, University of Texas Health San Antonio, San Antonio, TX USA; 3 Department of Population Health Sciences, University of Texas Health San Antonio, San Antonio, TX USA; 4 Department of Medicine, School of Medicine, UPMC Hillman Cancer Center, University of Pittsburgh, Pittsburgh, USA

**Keywords:** Adversarial attacks, Perturbation, Adversarial robustness, Medical image classification, Model complexity

## Abstract

**Background:**

Deep learning (DL) models are highly vulnerable to adversarial attacks for medical image classification. An adversary could modify the input data in imperceptible ways such that a model could be tricked to predict, say, an image that actually exhibits malignant tumor to a prediction that it is benign. However, adversarial robustness of DL models for medical images is not adequately studied. DL in medicine is inundated with models of various complexity—particularly, very large models. In this work, we investigate the role of model complexity in adversarial settings.

**Results:**

Consider a set of DL models that exhibit similar performances for a given task. These models are trained in the usual manner but are not trained to defend against adversarial attacks. We demonstrate that, among those models, simpler models of reduced complexity show a greater level of robustness against adversarial attacks than larger models that often tend to be used in medical applications. On the other hand, we also show that once those models undergo adversarial training, the adversarial trained medical image DL models exhibit a greater degree of robustness than the standard trained models for all model complexities.

**Conclusion:**

The above result has a significant practical relevance. When medical practitioners lack the expertise or resources to defend against adversarial attacks, we recommend that they select the smallest of the models that exhibit adequate performance. Such a model would be naturally more robust to adversarial attacks than the larger models.

## Background

Deep learning (DL) has achieved state-of-the-art performance in a variety of image classification tasks from natural image classification [1] to medical image analysis [2]. However, DL models are vulnerable to adversarial attacks—imperceptible input perturbations utilized to produce an incorrect model prediction [3]. This inherent weakness in DL poses a major security threat to medical DL models in that an attacker has the ability to alter the networks output. In fact, medicine may be uniquely susceptible to adversarial attacks [4].

Several defense techniques have been proposed to reduce model sensitivity to adversarial examples which include detection methods [5], defensive distillation [6], ensemble methods [7] and adversarial training [8]. Adversarial training is considered one of the most effective defense techniques. It minimizes the cost of a network trained on adversarial perturbations that maximize network error but suffers from performance degradation on unperturbed data [8]. Nevertheless, attaining adversarial robustness of deep neural networks remains an ongoing research effort.

DL has been extensively utilized in the medical domain. Several DL based medical devices and algorithms in healthcare have been approved by the FDA to assist in diagnosing disease such as HealthPNX, Critical Care Suite & SubtleMR [9]. In fact, DL models have achieved remarkable performance for chest x-ray [2], dermoscopy [10] and retinal fundus classification [11]. However, medical image based DL models are also vulnerable to adversarial attacks [4]. Adversarial attacks against healthcare systems could interfere with proper medical diagnosis and potentially cause misdiagnosis by imperceptibly altering medical imaging that serve as input to DL based medical devices and algorithms in healthcare. These modifications may result in erroneous medical treatment and fraudulent billing to healthcare insurance providers [4]. Patient treatment plans can be changed by attacking Electronic Health Records (EHR), which is the digital version of patient medical records [12]. Attackers can produce adversarial examples to generate a specific disease prediction from medical image DL models. In fact, universal adversarial perturbations can achieve misdiagnosis at a very low cost and high success rate [13]. Furthermore, medical image DL models are more vulnerable to adversarial attacks than natural image DNNs, i.e., adversarial attacks can succeed more easily on medical images using less perturbation [14].

Generally, in the case of natural images, larger models are considered to be more robust against adversarial attacks. In classical machine learning, the principle of Occam’s Razor suggests choosing simpler models as they are expected to generalize better; however, larger ImageNet architectures often produce state-of-the-art performance in natural image classification [15]. As a result, Occam’s Razor may not be a reliable heuristic for DL model selection in an adversarial setting. In fact, capacity is crucial for adversarial robustness [8], i.e., as capacity increases, natural image DL models become more resistant to adversarial attacks. Nevertheless, there is a trade-off between adversarial robustness and clean accuracy for natural image DL models [16]. However, the relationship between adversarial robustness and model complexity for medical image DL models has not been carefully studied.

DL models deployed in realistic clinical settings often employ large DL architectures such as Resnet [17] for medical image classifications. However, these large Resnets trained on medical images do not significantly exhibit greater performance than smaller models [18]. Instead, smaller, simpler models provide comparable performance to large overly complex networks for unperturbed medical images. In fact, model complexity may have contributed to the high vulnerability of medical image DL models [14]. This was primarily attributed to a sharp loss landscape that was hypothesized to be the result of a highly complex network for a simple classification task. Instead, we provide evidence that shows how model complexity influences adversarial robustness through decision boundary visualizations and saliency maps—image representation highlighting attention regions that influence a model’s output the most [19]. A recent study [13] found that model architecture did not play a significant role in adversarial robustness for medical image DL models against universal adversarial perturbations. However, they only evaluate performance on state-of-the-art DL architectures, which are considered to be over-parameterized for medical image classification.

In this paper, we investigate whether simpler DL models of reduced complexity can produce comparable or improved robustness to state-of-the-art large networks for medical image classification. With this in mind, we strive to understand “*How does model complexity impact adversarial robustness for medical image DL models*”? “*Could models of reduced complexity offer greater robustness for medical image DL* *models*”?. To this end, we investigate the role of model complexity in adversarial robustness for standard and adversarially trained medical image DL models. In summary, our contributions are as follows:

- Consider a set of medical image DL models that exhibit similar performances for a given task. These models are trained in the usual manner but are not trained to defend against adversarial attacks. We demonstrate that, among those models, simpler models of reduced complexity show a greater level of robustness against adversarial attacks than larger models that often tend to be used in medical applications.
- On the other hand, we also show that once those models undergo adversarial training, the adversarial trained medical DL models exhibit a greater degree of robustness than the standard trained models for all model complexities.

Our findings have a significant practical relevance. When medical practitioners lack the expertise or resources to defend against adversarial attacks, we recommend that they select the smallest of the models that exhibit adequate performance. Such a model would be naturally more robust to adversarial attacks than the larger models.

The remainder of this paper is organized as follows. In the Results section, we discuss adversarial robustness for medical image DL models of various complexity. In addition, we provide an interpretation on the role of model complexity through saliency maps and decision boundary visualizations. In the Methods section, we describe our experimental setup which includes details of our training method, attack methods, datasets and network architecture.

## Results

We evaluate robustness of the medical image DL models of 5 different complexities against adversarial attacks launched by FGSM and PGD. The magnitude of the perturbation was increased for each set of attacks to introduce more perturbation. To this end, we consider a model with the highest performance at a given $\epsilon$ to be more adversarially robust.

### Evaluation of standard trained models

**Fig. 1:**
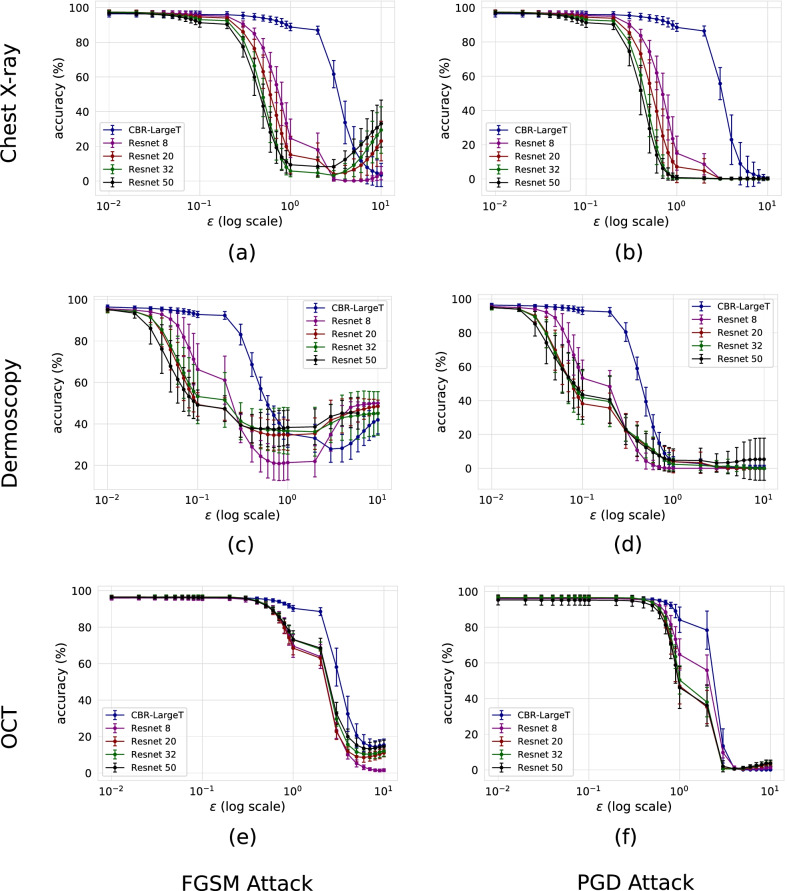
Average accuracy and standard deviation of FGSM and PGD attacks on medical image DNNs. Column 1 & 2 are FGSM and PGD attacks, respectively. Row 1, 2, & 3 are Chest X-ray, Dermoscopy & OCT datasets, respectively. All networks exhibit similar performance on unperturbed data for a given dataset. The magnitude of the perturbation was increased by $\epsilon$ ∈ [0*.*01*,*10]. Models of reduced complexity exhibit greater adversarial robustness

The average accuracy versus $\epsilon$ of medical image DL models are shown in Fig. 1 for both FGSM and PGD attacks. First, we notice that each of the standard trained models produced comparable performance on unperturbed data samples. Second, we observe an inverse relationship between model complexity and adversarial robustness for all medical image datasets. Particularly, the CBR-LargeT network is the least complex among all networks that were evaluated but it demonstrates the greatest robustness on all medical image datasets. In Table 1, the average accuracies of CBR-LargeT against PGD attacks were 88.37%, 92.63% and 78.35% for Chest X-ray, Dermoscopy, and OCT datasets, respectively. Similarly, Resnet8 exhibits greater adversarial robustness compared to Resnet50. This surprising behavior conflicts with the common belief that larger DL model are required to produce greater adversarial robustness [8]. We show that it is possible to attain greater robustness with a standard 5 layer CNN compared to state-of-the-art Resnet50 models for standard trained networks on medical image datasets. Taken together, these results suggest that among standard trained models that offer similar performance, medical image classifications could benefit more from less complex networks for adversarial robustness.

**Table 1:** Model performance at $\epsilon$ that produce the largest margin between least and most robust networks. Models of reduced complexity exhibit greater performance on perturbed medical images compared to larger, overly complex networks while maintaining comparable performance on unperturbed data

Attack	CBR-LargeT	Resnet-8	Resnet-20	Resnet-32	Resnet-50
**(a) Chest X-Ray Accuracy(%), **$\epsilon$** = 1**					
No Attack	96.43 + − 1.84	97.43 + − 1.01	97.46 + − 1.24	97.41 + − 0.98	96.90 + − 1.26
FGSM	88.83 + − 2.14	24.63 + − 11.12	15.13 + − 10.68	5.77 + − 3.30	9.37 + − 5.82
PGD	88.37 + − 2.31	15.07 + − 9.95	7.10 + − 9.26	0.43 + − 0.62	0.83 + − 1.23
**(b) Dermoscopy Accuracy (%), **$\epsilon$** = 0.1**					
No Attack	96.40 + − 0.95	95.53 + − 1.02	95.03 + − 1.16	95.07 + − 1.48	95.20 + − 0.85
FGSM	92.80 + − 1.48	66.30 + − 12.37	49.37 + − 5.70	53.23 + − 13.13	49.10 + − 7.28
PGD	92.63 + − 1.66	53.30 + − 10.61	38.17 + − 7.74	41.83 + − 15.77	43.63 + − 14.91
**(c) OCT Accuracy (%), **$\epsilon$** = 2**					
No Attack	96.30 + − 0.67	95.53 + − 1.08	95.03 + − 0.81	95.07 + − 1.05	95.20 + − 2.71
FGSM	88.58 + − 2.12	63.85 + − 6.56	63.00 + − 3.92	67.88 + − 3.94	68.65 + − 5.36
PGD	78.35 + − 10.70	55.85 + − 8.60	35.23 + − 9.30	37.98 + − 8.23	36.13 + − 11.56

### Evaluation of adversarial trained models

**Fig. 2:**
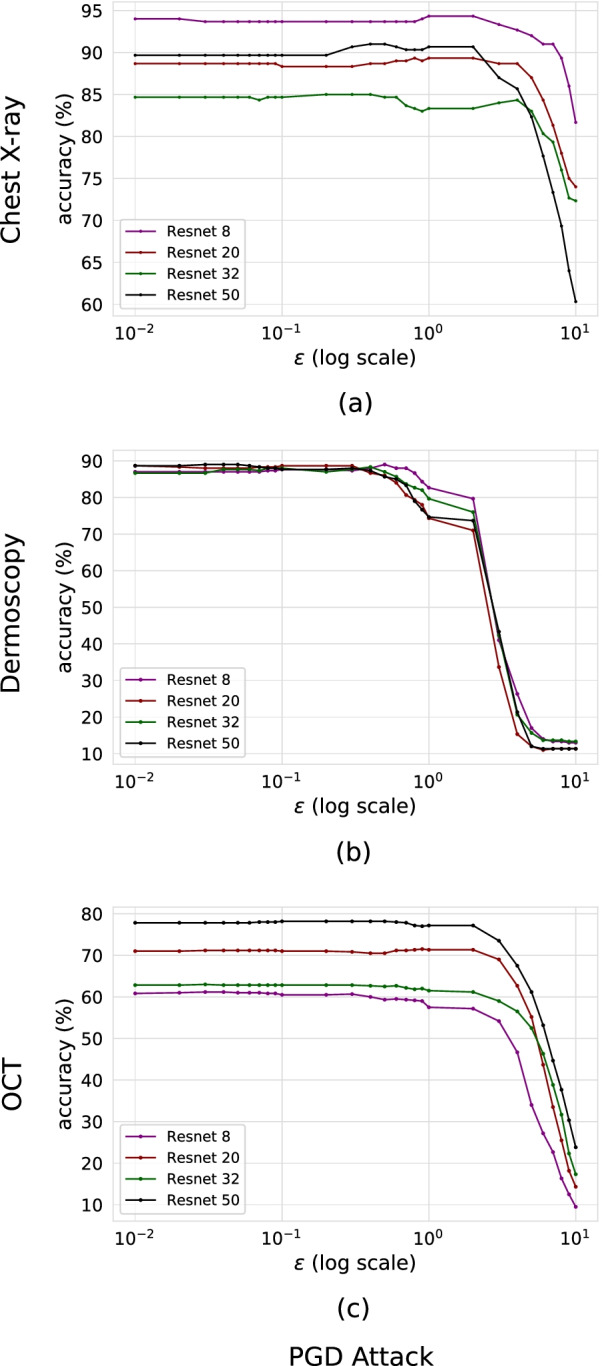
PGD adversarial training on Chest X-ray, Dermoscopy and OCT datasets, respectively. Chest X-ray and Dermoscopy models demonstrate greater robustness with Resnet8. OCT models provide greater robustness with Resnet50

The robustness of adversarial trained models was evaluated with adversarial examples generated using the PGD attack method. Figure 2 shows accuracy versus $\epsilon$ of adversarial trained models using PGD attack for Chest X-ray, Dermoscopy and OCT datasets. We observe an increase in robustness for all three datasets and a decrease in standard accuracy, i.e. performance on unperturbed images. The adversarial trained models outperform the corresponding standard trained networks by > 60%, > 73% & > 46% accuracy at $\epsilon$ = 10, 1 & 4 for Chest X-ray, Dermoscopy & OCT datasets, respectively. Table 2 highlights the adversarial trained model performance on unperturbed and perturbed images. The “No Attack” section of Tables 1 and 2 exhibit a drop in performance from standard to adversarial trained models on unperturbed images. This behavior is in line with the conclusion made by previous work that discovered a trade-off between accuracy and robustness, i.e. as models become more robust to adversarial examples they perform worse on unperturbed images [16].

**Table 2:** Adversarial trained model performance of perturbed and unperturbed data

Attack	Resnet-8	Resnet-20	Resnet-32	Resnet-50
**(a) Chest X-Ray Accuracy (%),**$\epsilon$** = 10**				
No Attack	94.01	88.27	84.92	89.90
PGD	82.14	74.05	72.96	61.11
**(b) Dermoscopy Accuracy (%),**$\epsilon$** = 1**				
No Attack	88.14	89.56	88.02	89.64
PGD	82.37	74.49	80.01	74.92
**(c) OCT Accuracy(%),**$\epsilon$** = 4**				
No Attack	78.12	71.01	62.77	88.67
PGD	47.28	62.56	57.06	67.95

In Fig. 2 we observe that adversarial trained models produce greater robustness at the cost of standard accuracy (accuracy on unperturbed images) compared to standard trained models shown in Fig. 1. This is especially true for the network trained with the OCT dataset shown in Fig. 2c, the Resnet 50 model produced the greatest standard accuracy and robustness. The models’ performance on unperturbed images is much higher than all other networks which resulted in greater robustness. The Chest X-ray adversarial trained model performance shown in Fig. 2a exhibits the opposite effect in that Resnet 8 produced the highest standard accuracy and it also provides the greatest robustness. The Dermoscopy adversarial trained models shown in Fig. 2b all exhibit similar standard accuracy but the Resnet 8 provides the greatest robustness. Based on our results, we conclude that for a given set of medical image adversarial trained models, the network with the highest standard accuracy will likely provide the greatest robustness. A previous study [20] has demonstrated that standard accuracy is correlated with robustness. On the other hand, if all networks provide similar standard accuracy then it is likely that the least complex network will provide the greatest robustness.

### Interpreting the role of model complexity in adversarial robustness

To understand why simpler networks were more adversarially robust than large overly complex networks, we analyzed the attention regions of saliency maps as model complexity increased. In addition, we visualize decision boundaries and adversarial example TSNE projections as model complexity increases.

#### Saliency maps

**Fig. 3:**
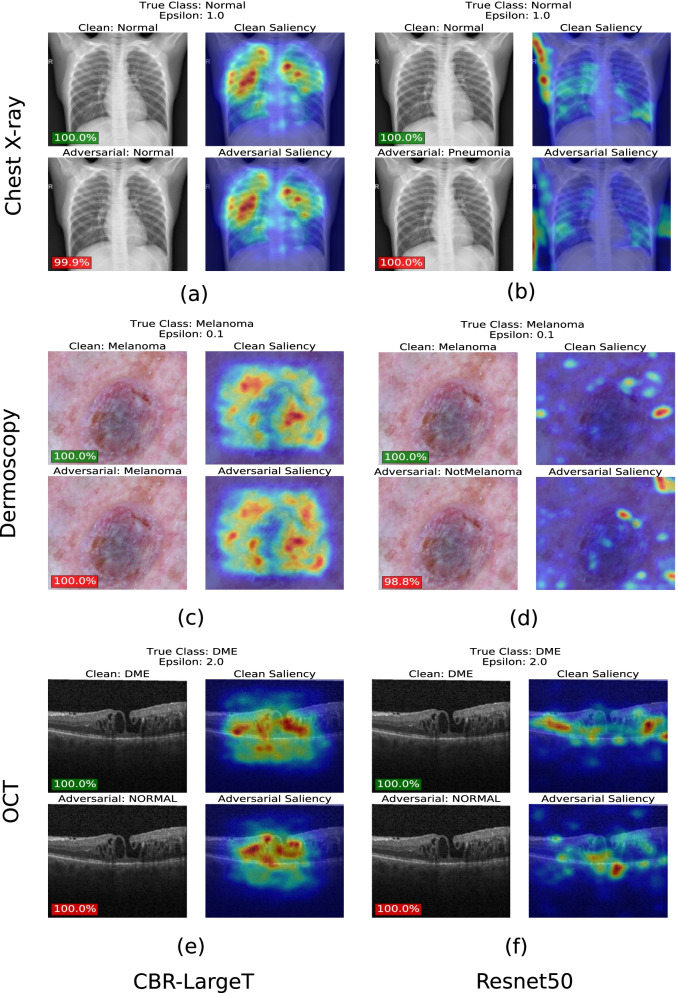
Saliency maps of standard trained CBR-LargeT (**a**, **c**, **e**) and Resnet50 (b, d, & f) for Chest X-ray, Dermoscopy & OCT datasets. Unperturbed images with predicted labels and corresponding saliency maps were visualized on row 1 (no attack) for (**a**–**f**), respectively. Imperceptibly perturbed images with predicted labels and corresponding saliency maps were visualized on row 2 (PGD attack) for (**a–f**), respectively. Saliency maps of CBR-LargeT are more concentrated on the regions of interest whereas Resnet50 includes attention regions that are more sporadic on areas that do not contribute to the classification of the disease

**Fig. 4:**
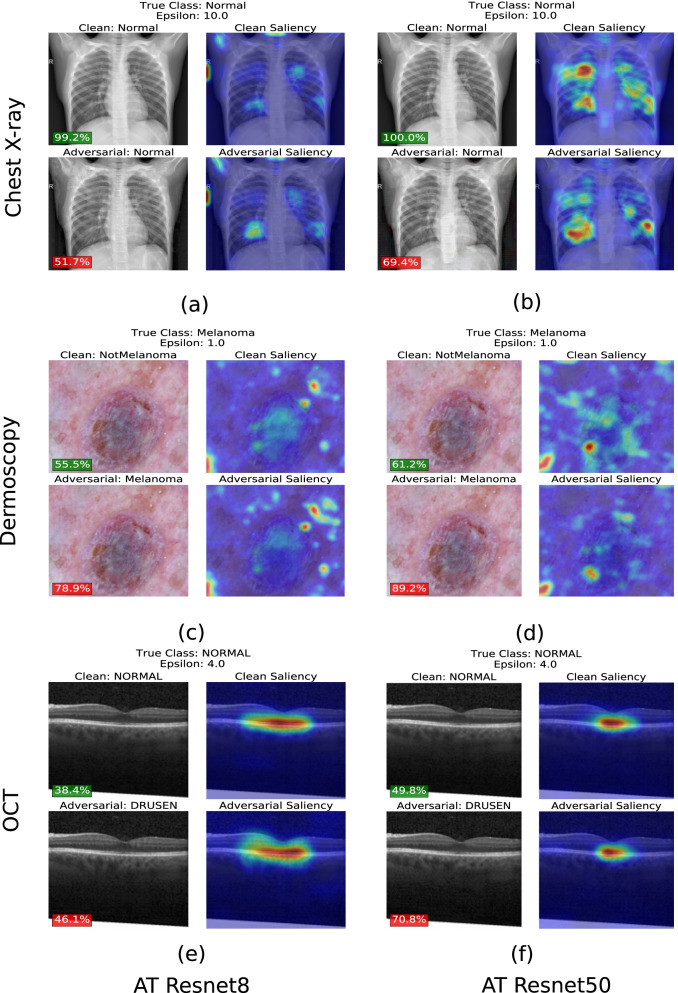
Saliency maps of adversarially trained (AT) Resnet8 (**a**, **c**, **e**) and Resnet50 (**b**, **d**, **f**) for Chest X-ray, Dermoscopy & OCT datasets. Unperturbed images with predicted labels and corresponding saliency maps were visualized on row 1 (no attack) for (**a–f**), respectively. Imperceptibly perturbed images with predicted labels and corresponding saliency maps were visualized on row 2 (PGD attack) for (**a–f**), respectively. Saliency maps of Resnet50 are more concentrated on the regions of interest whereas Resnet8 include attention regions that are more sporadic on areas that do not contribute to the classification of the disease

We first utilize saliency maps to understand why standard trained medical image DL models of reduced complexity produce greater adversarial robustness. We generate saliency maps for medical images before adversarial attacks where $\epsilon$ = 0 and after PGD attack where $\epsilon$ = 1, 0.1 & 2 for Chest X-ray, Dermoscopy & OCT datasets, respectively. In Fig. 3, we observe that the attention regions of CBR-LargeT are more concentrated on the regions of interest, whereas Resnet 50’s attention regions are spread out in regions that do not contribute to the classification of disease. For standard trained models, the CBR-LargeT network is the least complex and most robust model shown in Fig. 3 for all datasets which means that its performance does not change much with small perturbations. Consequently, the clean and adversarial saliency maps in Fig. 3a, c, e do not change much, it is the desired behavior. A previous study [21] reported that adversarial examples could be attributed to the presence of non-robust features (features that are weakly correlated with the true label) utilized by standard trained models which assign weight to features with non-zero correlation to obtain optimal performance. As a result, DL models learn to rely on non-robust features which adversarial perturbations can exploit causing major changes to the model output with small perturbations. Nonrobust features are considered useful since they contribute to a standard models’ ability to generalize with high accuracy, removing them would result in a reduction of standard accuracy. Another study [22] demonstrated that standard models trained with robust features (features that are strongly correlated with the true label) produce greater adversarial robustness. Robust features are useful and require larger perturbations to degrade model performance. In this paper, we demonstrate that CBR-LargeT can more accurately learn robust medical image features that are human perception aligned than Resnet 50. As a result, small adversarial perturbations rarely cause misclassification and adversarial saliency map attention regions are mostly unchanged when compared to clean saliency maps. Whereas, the Resnet 50 saliency maps in Fig. 3b, d, f are largely not human perception aligned considering that the strongly correlated features are mostly not significant for classification of the disease. These models do not seem to learn enough accurate robust features as is the case for CBR-LargeT which indicates that Resnet 50 learns more non-robust or weakly correlated medical image features. As previously stated, small adversarial perturbations to non-robust features can cause significant change to the model output. This can explain why some Resnet 50 adversarial saliency maps shown in Fig. 3 present very little change when compared to the corresponding clean saliency maps. We further examined the saliency maps of adversarial trained models in Fig. 4. We observe for all three datasets that adversarial training makes the attentions of especially more complex models focused. Now, the models focus within the anatomical regions contributing to the diagnosis rather than the anatomical regions of no interest or image background. This indicates that during adversarial training the model may help better identify the regions-of-interest that contribute toward correct classifications for both adversarial and clean data samples. Saliency map visualizations were implemented using keras [23]. We set the filter indices to the predicted class of a network for a given data sample.

#### Decision boundary

We next generate decision boundary visualizations of the standard and adversarial trained models. To generate the decision boundaries we utilized the output of the last fully connected layer. The features were utilized as input to t-distributed stochastic neighbor embedding (TSNE) [24] to reduce the input dimensionality and obtain a 2D projection of the data. Adversarial examples were generated from a subset of the test data for each $\epsilon$ and were combined with the entire dataset. We fit K-Nearest Neighbor classifiers (KNNs) [25] on the combined low dimensional data points (train, test, validate and adversarial examples) produced by the TSNE projection. KNN calculates the euclidean distance between data points and predicts a label based on how close new data points are to samples that the model stored when fitting the data. The KNN models were utilized to predict the class of each point on the decision boundary visualizations which were implemented using the mlxtend library [26].

**Fig. 5:**
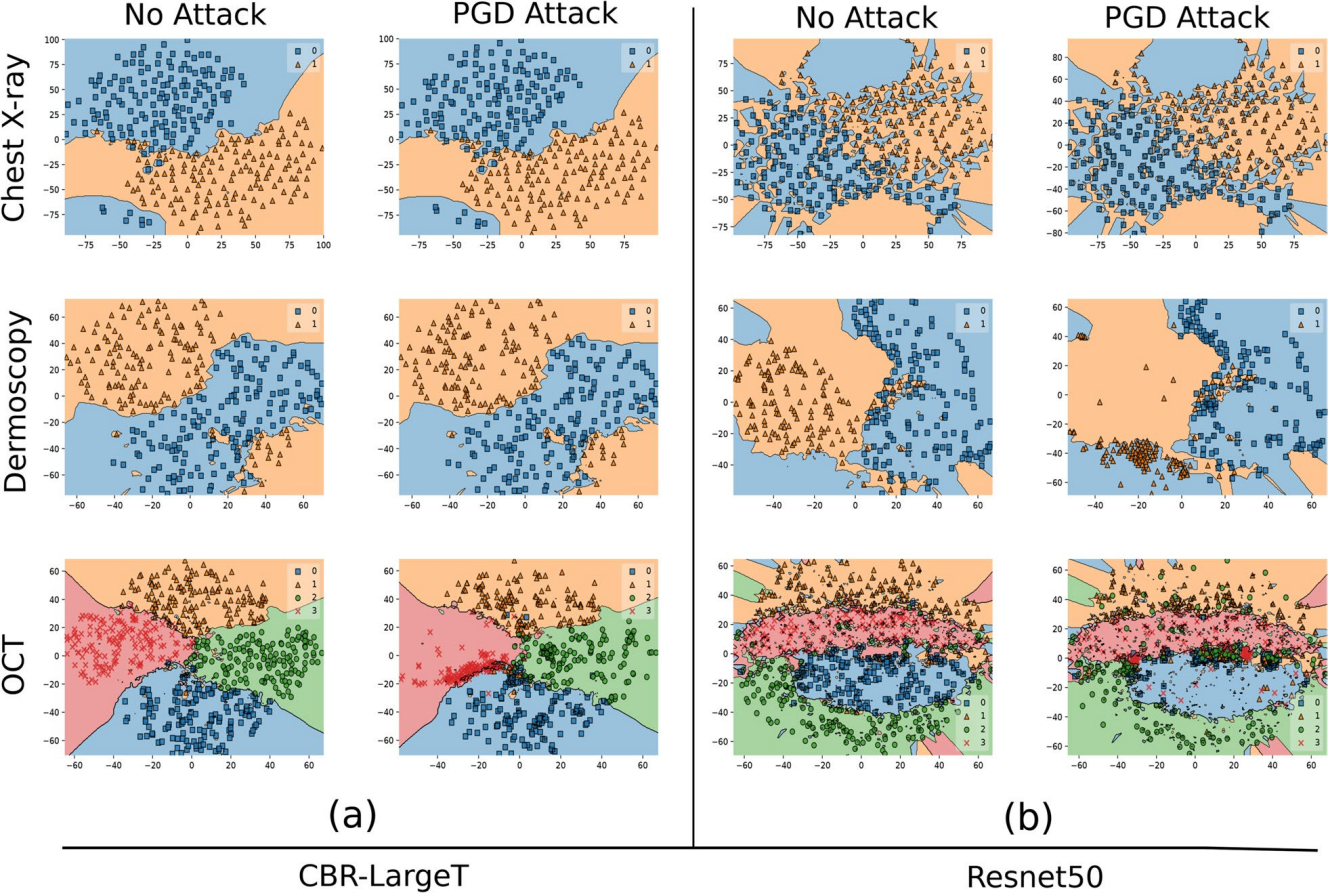
Decision boundary projection of standard trained CBR-LargeT and Resnet50 for Chest X-ray, Dermoscopy & OCT datasets. Unperturbed samples were projected on column 1 (no attack) and perturbed samples were projected on column 2 (PGD attack) for (**a**, **b**), respectively. The more complex decision boundary in (**b**) resulted in samples that were closer to the decision boundary in the projected space which increased medical image DNN sensitivity to input perturbations

**Fig. 6:**
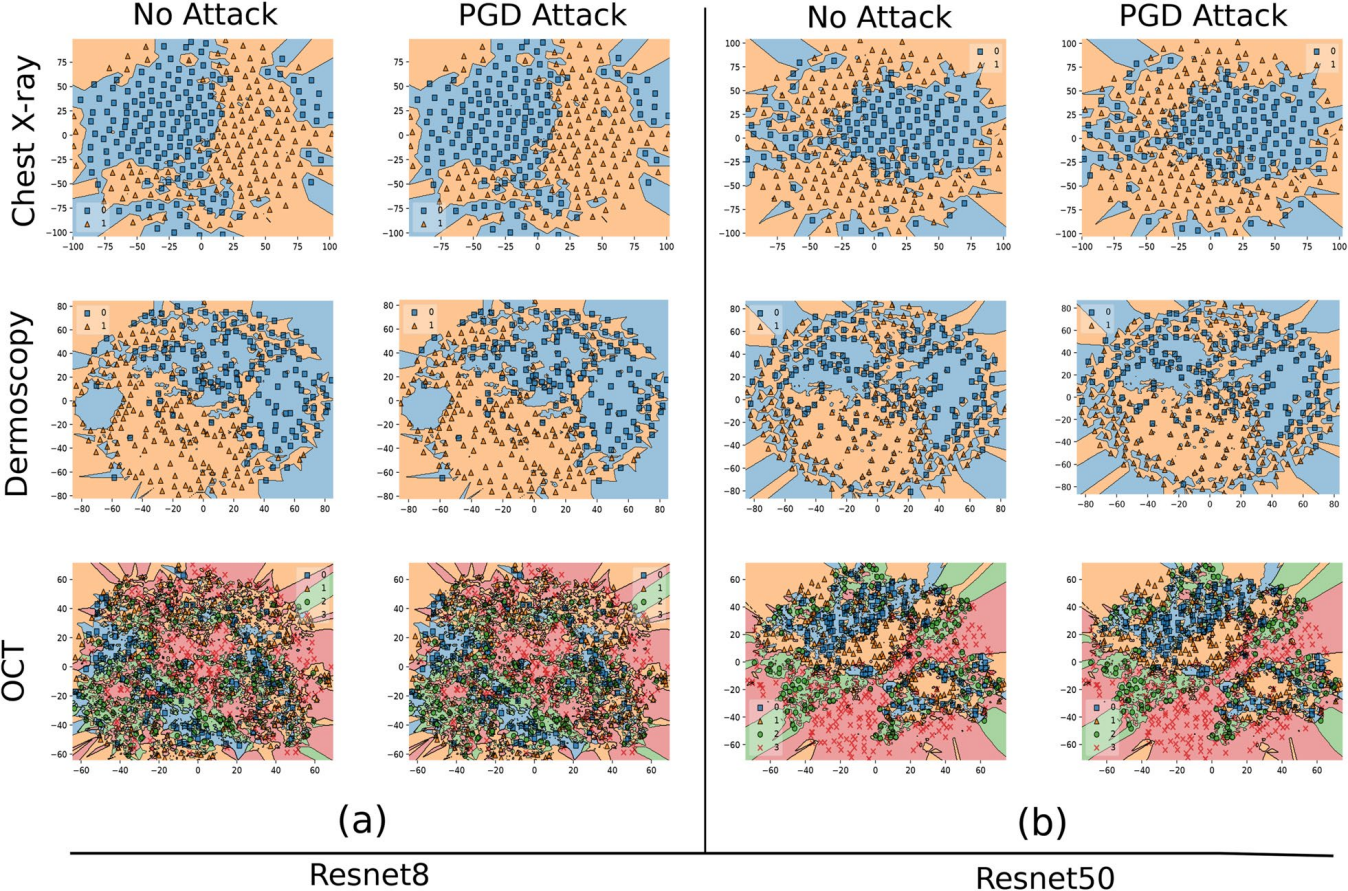
Decision boundary projection of adversarially trained (AT) Resnet8 and Resnet50 for Chest X-ray, Dermoscopy & OCT datasets. Unperturbed samples were projected on column 1 (no attack) and perturbed samples were projected on column 2 (PGD attack) for (**a**, **b**), respectively. The complexity of adversarial trained decision boundaries increased compared to the corresponding standard trained model decision boundary in Fig. 5

We visualize the decision boundary for CBR-LargeT and Resnet50 before adversarial attacks where $\epsilon$ = 0 and after PGD attack where $\epsilon$ = 1, 0.1 & 2 for Chest X-ray, Dermoscopy & OCT datasets, respectively. In Fig. 5b, we observe that data points cluster together more tightly along the decision boundary for Resnet 50 models as opposed to Fig. 5a, where the data points are sparsely projected across the boundary for CBR-LargeT. The Resnet 50 decision boundary is more complex and results in data samples that are closer to the decision boundary in the projected space, which increases medical image DL models’ sensitivity to input perturbations. Although Resnet 50 and CBR-LargeT consistently produced comparable performance on multiple subsets of the train and test datasets for unperturbed medical images, it is evident that CBR-LargeT provides greater adversarial robustness as projected data samples are much further from the decision boundary. Large state-of-the-art DL models are overly complex for medical image classification, which result in highly sensitive networks that are more vulnerable to small adversarial perturbations as projected data points are closer to the decision boundary. In contrast, per Fig. 6 of decision boundaries from adversarial trained models, we observe that adversarial training in general produces more complicated decision boundaries for all three datasets. We also observe that the closer the data points are to the decision boundary edges the more vulnerable the network since it is less confident and small perturbations are able to easily increase the correlation of non-robust features toward another target class. This is evident in Fig. 6a for OCT dataset, Fig. 6b for Chest X-ray and Fig. 6b Dermoscopy datasets.

## Discussion

We showed that highly complex medical image DNNs are more vulnerable to adversarial attacks than models of reduced complexity. A previous study [14] hypothesized that model complexity may contribute to the robustness of medical image DNNs but they only show that medical image DNNs have a sharp loss landscape compared to natural image DNNs. In this study, we demonstrated that smaller, simpler medical image DNNs provide greater adversarial robustness. Typically, larger DNNs are considered more robust for natural image classification, however, medical image DNNs do not require large state-of-the-art networks for optimal performance. Adversarial attacks can succeed more easily as model complexity increases for medical images. Standard trained DL models that perform well on ImageNet data are not guaranteed to provide adversarial robustness. In fact, large state-of-the-art DNNs are overly complex for medical image classification and result in data samples that are closer to the decision boundary as seen in the projected space which increase the sensitivity of medical image DNNs to input perturbations. Although, the high complexity of DL model decision boundaries cannot be fully captured with current methods, due to the loss of information in dimensionality reduction, the decision boundary projection visualizations do provide insight into why our models demonstrate such sensitivity to small perturbations. This was also shown using saliency maps by visualizing how the attention regions changed as the model was under attack. As a guidance on model selection for a given set of standard trained medical DL model candidates we suggest that practitioners first evaluate the performance of each network on unperturbed medical images to realize networks of comparable performance and select the least complex model among the realized networks to produce the greatest robustness against adversarial attacks.

## Conclusions

In our study, we investigated the role of deep learning model complexity in adversarial robustness for medical images and demonstrated that standard trained medical image DL models of reduced complexity are more robust to adversarial attacks than large overly complex networks. We show that medical image DL models are more adversarially robust as model complexity decreases. Our saliency map visualizations reveal that standard trained models of reduced complexity learn the features that contribute to the classification of disease better. The decision boundary visualizations show that larger overly complex networks result in data samples that are closer to the decision boundary in the projected space which increase the sensitivity of medical image DL models to input perturbations. We therefore recommend deep learning practitioners in the medical community to first evaluate the performance of a given set of DL models candidates on unperturbed medical images to realize networks of comparable performance and select the least complex model among the realized networks to produce the greatest robustness against adversarial attacks.

## Methods

### Medical image datasets

In this work, we use three publicly available medical image datasets to study adversarial robustness, which include Chest X-Ray, Dermoscopy and Optical Coherence Tomography (OCT). The chest x-ray dataset [27] consists of 5,863 grayscale chest radiograph images used to diagnose thorax disease. It includes two classes, where each image is labeled as “Pneumonia” or “Normal”. The dermoscopy dataset [28] contains 17.8 K color images of skin lesions, which are used to diagnose melanoma skin cancer. It includes two classes, where each image is labeled as “Melanoma” or “NotMelanoma”. We consider all non-melanoma images to be part of the NotMelanoma class [29]. The OCT dataset [27] consists of 84,495 grayscale images with four classes—including “Choroidal Neovascularization (CNV)”, “Drusen”, “Diabetic macular edema (DME)”, and “Normal”. It utilizes light waves to take cross-section imagery of the retina to assist in diagnosing retina disease and disorders in the optic nerve.

### Medical image deep learning models

Typically, large state-of-the-art ImageNet architectures such as Resnets are utilized for medical image classification [30]. However, a recent study [18] found that simpler architectures such as CBR-LargeT provide comparable performance to large ImageNet architectures on unperturbed medical images. To this end, we evaluate the role of model complexity using a family of four Resnet architectures and a five-layer Convolutional Neural Network (CNN). Resnets are large state-of-the-art DL architectures that consist of several blocks of residual modules and skip connections [17]. We adjust the complexity of the network by reducing the amount of residual modules and skip connections. The Resnet architectures included in our study are: Resnet50, Resnet32, Resnet20 and Resnet8. The CBR-LargeT architecture is a standard CNN that consists of five convolution layers, initially each layer has 32 filters and a 7 × 7 kernel size. The amount of filters are doubled at each convolution layer while the kernel size remains constant for all layers. All convolution layers are followed by batch normalization, ReLu activation and a max pooling layer with 3 × 3 window and 2 × 2 stride. All networks utilize softmax activation at the output layer.

### Training procedure

We initiated training of each DL model with random initialization of model parameters as a previous study [18] demonstrated that utilizing pretrained ImageNet weights (transfer learning) for medical image DNNs did not significantly improve model performance. We used the Adam optimizer with a batch size of 32 and a learning rate scheduler. Checkpoints were utilized to store the model with the highest validation accuracy during the training procedure. All medical images were resized to 224 × 224 and normalized between 0 and 1. Each dataset was randomly shuffled and split ten times to generate multiple subsets of the train, test and validation set. Each network was trained ten times for a given dataset to assess the average performance of all models across multiple subsets of the data.

### Adversarial attack methods

In this section, we provide an overview of adversarial attack methods utilized to generate adversarial examples. The attack methods include L-BFGS, Fast Gradient Sign Method, One-Step Target Class Method, Basic Iterative Method and Projected Gradient Descent Method [31]. The fast gradient sign method [32] is a fast and easy way to generate adversarial examples while the projected gradient descent method [8] is one of the strongest adversarial attack methods. In this study, we employ both methods using the least likely class as they are commonly utilized to evaluate the robustness of deep neural networks.

### Fast gradient sign method

FGSM is a max-norm constrained adversarial attack method that solves for the perturbation that maximizes the classification loss [32]. This method is a single step attack, which perturbs the image in a single step as

1 $$x^{adv} = x + \epsilon sign\left( {\nabla_{x} L\left( {\theta ,x,y_{true} } \right)} \right)$$

where $\epsilon$ is the magnitude of the perturbation which constrains the amount of perturbation allowed in each pixel of an image, *x*^*adv*^ is the perturbed adversarial sample, (L(·)) is the classification loss function, ∇_*X*_L is the gradient with respect to the unperturbed sample (*x*), *θ* is the DL model weights, and (*y*_*true*_) is the true label.

#### One-step target class methods

The One-Step Method is an extension of FGSM that maximizes the probability of a specific target label that is not likely to be the true label for a given input sample. The goal is to solve for a perturbation that minimizes the cost function for the true label and the target label [33]. This method perturbs the input image as

2 $$x^{adv} = x - \epsilon sign\left( {\nabla_{X} L\left( {\theta ,x,y_{target} } \right)} \right)$$

where $\epsilon$ is the magnitude of the perturbation which constrains the amount of perturbation allowed in each pixel of an image, *θ* is the DL model weights, *x*^*adv*^ is the perturbed sample of a single iteration, ∇_*X*_L(*θ,x,*^*y*^_*target*_) is the gradient (∇_*x*_) of the loss function (L(·)) with respect to the input data sample (*x*) and target label (*y*_*target*_).

#### Least likely class method

The Least Likely Method utilizes the least likely predicted class of a trained network for a given data sample to generate an adversarial example [34]:

3 $$y_{LL} = \mathop {\arg \min }\limits_{y} \left\{ {p\left( {y_{true} |x} \right)} \right\}$$

where argmin_*y*_{*p*(*y*_*true*_|*x*)} is the minimum probability (*p*) of the true label (*y*_*true*_) for a given data sample (*x*).

### Projected gradient descent method

PGD is one of the strongest first-order attack methods and is an extension of FGSM. It iteratively attempts to produce an optimal perturbation from a random point within an L-∞ ball, which defines a space around the original data point that has a radius normally equivalent to epsilon [8]. PGD iterates as follows:

4 $$x^{t + 1} = \mathop{\prod}\limits_{x + s} (x^{t} + \alpha sign(\nabla_{x} L(\theta ,x,y_{target} )))$$

where *x*^*t*^ is the adversarial example at the t-th iteration, ^Q^(·) is the projection function to project adversarial examples back onto the L-∞ ball after each iteration, *α* is the step size and *θ* is the DL model weights.

### Generating adversarial examples

In our study, we generate adversarial examples with targeted FGSM and PGD attacks using the least likely class method for the target label. The magnitude of the perturbation was increased by $\epsilon$ ∈ [0*.*01*,*10] for each set of attacks. In addition, for PGD attacks we utilize 20 iterations with a step size *α* = ($\epsilon$ ∗ 0*.*1) for each attack and corresponding epsilon. Adversarial attacks can be deployed in a white-box or black-box attack setting. In the black-box attack setting the attacker has zero knowledge of the training data, architecture or model parameters. In the white-box attack setting the attacker has full knowledge of the target system, i.e., the attacker knows the training data, architecture and model parameters. The white-box attack setting allows security practitioners to perform a worst-case evaluation of the deep learning model under attack. To this end, we focus on a white-box attack setting as the source architecture and model parameters were known and utilized to generate adversarial examples. Adversarial examples were generated with a subset of the test data that the model was not previously exposed to during training and validation. Approximately, 150 data samples were randomly selected from each class of the test set to generate adversarial examples without data sample replacement. The FGSM and PGD attacks were implemented using the Cleverhans library [35].

### Adversarial training

Adversarial training was first introduced in 2015 [32], wherein they included adversarial examples into the training procedure to generate robust models. However, these trained models were still vulnerable as model robustness is directly related to the strength of adversarial samples being used during training. To address this in 2017, a new adversarial training algorithm that uses multi-step based PGD adversaries was proposed [8]. This achieves state-of-art robustness against L-infinity attacks on MNIST and CIFAR-10 dataset. A min–max formulation was used in training DL models [8]:

5 $$\mathop {{\text{min}}}\limits_{\theta } \rho (\theta ),\quad {\text{where}}\,\,\rho (\theta ) = {\mathbb{E}}_{(x,y) \sim {\mathcal{D}}} \left[ {\mathop {{\text{max}}}\limits_{\delta \epsilon {\mathcal{S}}} L(\theta ,x + \delta ,y_{target} )} \right]$$

where min_*θ*_* ρ*(*θ*) represents the classification task, E(_*x,ytarget*)_ represents the empirical loss on the sample distribution *p*_*x,ytarget*_. The above saddle-point formulation is a composition of inner maximization and outer minimization problem. The former aims to find an adversarial version of *x*, using Eq. (4), to provide high adversarial loss, while the latter attempts to find model parameters *θ* to minimize the empirical classification loss. A previous study [8] found that robustness against PGD adversary provides robustness against all first-order adversaries and DL models with larger capacity can fit adversarial samples better. Motivated by the model performance using Eq. (5) on computer vision datasets, in this study we aim evaluate the performance of medical DL models using Eq. (5) against adversarial and clean samples across different model capacities.

In our study, ResNet architectures of varying capacities—8, 20, 32, 50 layers were trained to generate adversarial trained models. The final layer for all the models were softmax with two neurons for Chest X-ray and Dermoscopy datasets, and four neurons for the OCT dataset. The networks were trained against adversarial perturbations that are max norm bounded. Each model was trained using initial weights from standard training of its counterpart network capacity, with learning rate of 0.001 and trained until the loss of the network would not further reduce or increase accuracy. To generate attacks during adversarial training, $\epsilon$ was set to 3/255, 1/255 and 10/255, with the step size set to $\epsilon$/10 and perturbation steps of 7, 5 & 5 for Chest X-ray, Dermoscopy & OCT datasets, respectively.

## Data Availability

The datasets generated and/or analysed during the current study are available in the GitHub repository, github.com/drodriguez3/MedicalDLM-Complexity-AdvRobust.

## Abbreviations

DNNs: Deep neural networks
Resnet: Residual network
OCT: Optical coherence to-mography
CNV: Choroidal neovascularization
DME: Diabetic macular edema
CNN: Convolutional neural network
CBR-LargeT: Conv2d-Batchnorm-Relu-Large-Tall
FGSM: Fast gradient sign method
PGD: Projected gradient de-scent
TSNE: T-distributed stochastic neighbor embedding
KNN: K-nearest neighbor
